# A Pandemic Instrument Can Start Turning Collective Problems into Collective Solutions by Governing the Common-Pool Resource of Antimicrobial Effectiveness

**DOI:** 10.1017/jme.2022.75

**Published:** 2022

**Authors:** Isaac Weldon, Kathy Liddell, Susan Rogers Van Katwyk, Steven J. Hoffman, Timo Minssen, Kevin Outterson, Stephanie Palmer, A.M. Viens, Jorge Viñuales

**Affiliations:** 1: YORK UNIVERSITY IN TORONTO, CANADA; 2: UNIVERSITY OF CAMBRIDGE, CAMBRIDGE, UK; 3: UNIVERSITY OF COPENHAGEN, COPENHAGEN, DENMARK; 4: CARB-X, BOSTON, MA, USA; 5: BOSTON UNIVERSITY, BOSTON, MA, USA

**Keywords:** Antimicrobial Resistance, Common-Pool Resources, Equity, Governance, Collective Action

## Abstract

To address the complex challenge of global antimicrobial resistance (AMR), a pandemic treaty should include mechanisms that 1) equitably address the access gap for antimicrobials, diagnostic technologies, and alternative therapies; 2) equitably conserve antimicrobials to sustain effectiveness and access across time and space; 3) equitably finance the investment, discovery, development, and distribution of new technologies; and 4) equitably finance and establish greater upstream and midstream infection prevention measures globally. Biodiversity, climate, and nuclear governance offer lessons for addressing these challenges.

Literature on antimicrobial resistance (AMR) has grown enormously in the last 10 years. While this growth is welcomed, the sudden rise in AMR literature presents a challenge for time-pressured policymakers. Specifically, it makes it difficult to gather and grasp all the necessary information, concepts, and controversies relevant for devising policy solutions for AMR. This plight is increasingly cumbersome in the context of global pandemic instrument negotiations, where negotiators must quickly identify the sum of issues that fit within a pandemic instrument and design strategies to effectively resolve these issues. In this paper, we present two tools from social science that treaty negotiators can leverage to identify the relevant governance challenges associated with AMR and design a pandemic instrument that incorporates effective solutions to address this urgent threat. The first is a problem synthesis framework that outlines the range of global governance problems around antimicrobial resistance. The second includes select examples where collective action theory has been concretely applied to address other global common pool resources similar to AMR, namely, biodiversity, climate, and nuclear governance. These examples can help signal toward potential global solutions for AMR.

## 1. Problem Synthesis: AMR Governance Challenges

Social science research reveals a long catalogue of social challenges associated with the rise of global antimicrobial resistance. With the help of this first tool, we translate these social problems into tangible and discrete governance challenges that must be addressed at the global level (Table 1). These specific governance challenges are behavioral and structural targets for global policy intervention that should be addressed by the pandemic instrument.

In this paper, we present two tools from social science that treaty negotiators can leverage to identify the relevant governance challenges associated with AMR and design a pandemic instrument that incorporates effective solutions to address this urgent threat. The first is a problem synthesis framework that outlines the range of global governance problems around antimicrobial resistance. The second includes select examples where collective action theory has been concretely applied to address other global common pool resources similar to AMR, namely, biodiversity, climate, and nuclear governance. These examples can help signal toward potential global solutions for AMR.

**Table 1:** Summary of Governance Challenges

	Social Aspects	Governance Challenges	Equity Challenges	Solutions under The Pandemic Treaty
**Access**	Improving access to antimicrobials is an urgent social challenge as millions of people around the world, mostly in low-income settings, still do not have access to effective antimicrobial medicines (2).	Places that have access will not necessarily act to help those who do not have access unless they recognize either a moral imperative to act or an enlightened self-interest. Places without access are not well positioned to improve access on their own.	Global distribution of disease burden, access to antimicrobials, and wealth is uneven and places without access tend to be LMICs, rural areas, and already marginalized groups.	A pandemic instrument should equitably address the access gap for antimicrobials, diagnostic technologies, and alternative therapies including other medical countermeasures.
**Conservation**	Every use, regardless of the setting, potentially accelerates AMR. Use across many sectors for various purposes is driven by deeply engrained structures and incentives.	Circulation of AMR bugs across political boundaries means that conservation is only effective if many countries do it. This factor disincentives individual countries from acting because the expense of such action will disadvantage them and not work unless other countries act as well.	Some populations have greater clinical need of antimicrobials now, plus there are uneven histories of use between high- and low-income countries.	A pandemic instrument should equitably conserve antimicrobials to sustain effectiveness and access across time and space.
**Innovation**	New drugs and technologies are not being developed fast enough to keep pace with rising resistance.	Investing in R&D imposes individual costs for social benefits.	Who sets the priorities for investments in innovation? Who innovates and thus benefits from profit-margins and R&D infrastructure development? There is a risk that new antimicrobial drugs will not be accessible in low-income country markets due to costs, scarcity, or supply/distribution challenges.	A pandemic instrument should equitably finance the investment, discovery, development, and distribution of new technologies including new antimicrobials and other medical countermeasures.
**Infection Prevention**	Lack of upstream measures and midstream measures, overarching global coordination frameworks, standardised global surveillance systems, and integrated One Health solutions (especially for environmental AMR).	It is not fair to expect that LMICs can improve their IP without external funding. An alternative framing: richer countries and pharmaceutical companies *could* do more to improve global IP including through global resource redistribution but choose not to.	Histories of global exploitation and colonialism have stymied IP investments and improvements in LMICs. Globally disadvantaged groups are more likely to face health risk (e.g., women, refugees, migrants, rural, remote, racialized, poor, and marginalized groups).	A pandemic instrument should equitably finance and establish greater upstream and midstream infection prevention and control measures globally.

**Table legend:**

**Column 1** identifies the four governance areas of access, conservation, innovation, and prevention

**Column 2** describes the social aspects of each of these four areas

**Column 3** describes the governance challenges associated with these four areas

**Column 4** describes the transversal issue of achieving equity, which affects each of the four areas of access, conservation, innovation, and IP; Equity cannot be after thought but must be built into every AMR policy across these 4 governance areas.

**Column 5** presents a guiding principle for each governance area, which can help determine whether a pandemic instrument is adequately addressing AMR

We find that AMR governance challenges fall into four broad areas: (1) improving access to antimicrobials, diagnostic technologies, and alternative therapy; (2) conserving the effectiveness of existing antimicrobials; (3) spurring innovation for antimicrobials, diagnostic technologies, and alternative therapies, as well as new behavioral, policy, and economic techniques; and (4) bolstering global infection prevention measures.^1^

These areas accentuate existing inequities that must be overcome by any policy intervention, making a fifth governance area, the challenge of achieving equity, a transversal issue that cuts across the first four areas. For each of the four areas of access, conservation, innovation, and prevention, we describe the social aspects of the problem, emphasize the global governance issues that persist within them, examine the equity considerations that each area presents, and, finally, prescribe an AMR specific principle to guide how the pandemic instrument should addresses AMR (Table 1).

### 1.1 Access

For many, the most urgent challenge remains the fact that millions of people around the world, mostly in low-income settings, still do not have access to effective antimicrobial medicines.^2^ These places face an unjust and somewhat paradoxical triple burden of a lack of access to effective antimicrobials, a high burden of infectious disease, and the highest burden of AMR-related mortality and morbidity.^3^ A lack of access to antimicrobials may accelerate AMR by enabling infections to spread and forcing people to seek substandard drugs, extend their courses and doses, or take smaller doses to share scarce antimicrobials among family.

The governance challenge for access is two-fold. *First*, there are few measures available for those who face the greatest access challenges to improve their situation due to ongoing legacies of colonialism, commercial determinants of health, and current global systems of exploitation.^4^ For example, previous studies have demonstrated that several important medicines are not even registered with national regulatory bodies in many LMICs for sale or distribution.^5^ These settings are often overlooked by companies’ access strategies for various reasons, including supply chain issues, lack of perceived market viability, and unfavorable government regulations like price policies.^6^ *Second,* actors with access to effective antimicrobials (e.g., high-income countries and pharmaceutical firms) are unlikely to act and help those without access unless they recognize a compelling reason or obligation to do so. A sufficiently strong motivation or obligation might arise from a sense of justice, whether directly (as a moral imperative or from international pressure) or indirectly (because of bottom-up political demand), international law, or, alternatively, an enlightened self-interest. The possible connection with domestic interests is that global health security from infectious disease can only be achieved when all have basic minimum access to antimicrobials.

### 1.2 Conservation

Conserving antimicrobial effectiveness for current and future generations remains a major problem.^7^ Antimicrobials are still used widely across sectors for various purposes. In human health settings, antimicrobials are used to treat deadly infections and enable many life-saving procedures, such as surgery and chemotherapy.^8^ In agriculture, the same antimicrobials are used to treat and prevent infection, improve yields, and promote growth. The problem is that each use, regardless of the setting or purpose, potentially accelerates AMR. Meanwhile, antimicrobial runoff through waste disposal accelerates AMR in the environment. Thus, there is an urgent need to ensure that appropriate antimicrobials are only used when important and essential. By revisiting some of the ways in which antimicrobials are currently used and distributed, conservation policies could significantly reduce AMR.^9^

This involves big challenges, however. Some highly entrenched uses of antimicrobials — in multiple sectors — would need to change for conservation policies to be successful. Another obstacle for enacting conservation measures is that effects will not be noticeable unless many countries implement them at the same time, which means that tackling overuse in a country may make little difference overall if other countries are not doing the same. Three further factors compound this governance challenge. First, antimicrobial use is driven by deeply entrenched structures and incentives meaning that it is costly and difficult to transform the relevant social practices.^10^ Second, this first point means conservation is not something that individuals can accomplish themselves, but rather must be an intentional, collective societal effort at the national level.^11^ Third, adopting conservation efforts will impose several immediate tangible costs on those who implement them, including potentially making their own economies less competitive, with only the promise of a less tangible social benefit in the future, and no guarantee that others will adopt similar initiatives.^12^

### 1.3 Innovation

Innovation for new antimicrobials is plagued with several systemic market failures. Pharmaceutical firms feel poorly incentivized to develop new drugs because the market for last resort antimicrobials is by definition small (the drug is designed to be used in exceptional cases). That means that new antimicrobials are not being developed fast enough to replace those to which key pathogens are becoming resistant.^13^ Similarly, there remains a lack of innovation for both diagnostic technologies and alternative therapies. Diagnostic technologies help to facilitate accurate and swift diagnosis of infections, their susceptibility to available drugs, and, therefore, more precise prescribing practices. Meanwhile, alternative therapies could act as substitutes for antimicrobials. The world also needs innovation for new social, political, and behavioral insights to address AMR. Such insights could include new ways to nudge people toward more appropriate use, or even new forms of plants that are more resilient to infection or agricultural practices that reduce the demand for antimicrobials in the first place.^14^

The current lack of investment in antimicrobial research and development is driven by a mismatch between their market and social value. One could also argue that despite the lack of economic incentives to innovate, there is a moral imperative for pharmaceutical companies to develop new drugs and expand access, given their position to do so, the high social value of antimicrobials, and the history of uneven use across countries of different income levels.

A range of market considerations further exacerbate the innovation challenge including the high costs and failure rates for drug development, the possibility that new antimicrobials may reduce markets for existing antimicrobials, the fact that new drugs are only needed in case of current antimicrobial failure, and the fact that only one course of antimicrobials is typically needed per treatment.^15^ Furthermore, markets for antimicrobials can be further restricted by stewardship efforts to conserve use. These economic factors mean that for antimicrobial research and development, there is the same high business risk as other pharmaceutical development initiatives, but with a lower likelihood of reward, except in crisis situations (e.g., a pandemic caused by an antimicrobial resistant pathogen). In crisis situations, however, access and equity considerations become particularly challenging, as shown by access to COVID-19 vaccines in the face of debates about intellectual property rights.

### 1.4 Infection Prevention

Antimicrobials are so widely used across sectors that they are essentially like invisible infrastructure that cover up shortcomings in infection prevention measures.^16^ Improving infection prevention (IP) is of special importance as it would reduce the risk of infection in the first place and, thereby, the demand for antimicrobial use.^17^ Yet, several upstream IP measures are missing globally, not the least of which is funding for improving health systems and social welfare in low-income settings.^18^ Examples of upstream measures that could help reduce AMR include addressing the root causes of infections and outbreaks and improving overall living and working conditions globally, while also addressing the conditions through which refugees, migrants, and other displaced people seek safety. Midstream measures, on the other hand, could include efforts to improve sanitation infrastructure, animal husbandry practices, and medical protocols, which could all help to curb infections and thereby AMR.

Another challenge for IP is AMR surveillance.^19^ Globally, there is a lack of overarching coordination frameworks across different levels of social organization (i.e., global, regional, national, sub-national) and across different sectors of human activity (i.e., human health, trade, and food). Without a standardized surveillance framework, it is impossible to track and compare human, animal, and environmental practices, as well as the evolution of infections, resistance, and their spread.

The governance challenges for improving IP in this context are similar to those for improving access. It is not fair to expect, those without the current capacity to improve their IP to do so without external help. Perhaps, a better way to formulate this challenge is that high-income countries and pharmaceutical firms could, in earnest, do more to improve global IP including through global wealth and medicine redistribution, but they lack (or fail to see) the incentive to do so. To reiterate, achieving a more equitable approach to global investments in IP would either require strong moral compasses in places with the capacity to intervene, or the recognition of shared global interdependence and vulnerability to infectious disease.

### 1.5 Challenges for Achieving Equity

Equity is a major challenge in all matters of global health and represents a significant AMR governance issue. AMR simultaneously acts as a prism that reveals many existing global inequities and a multiplier that deepens and exacerbates them. Fundamentally, equity has to do with the distribution of things of value (e.g., assets, opportunities, and outcomes) and in this way, all AMR policies must recognize how interventions may reproduce or worsen global health inequities.

Considerations for improving IP must recognize that lower- and middle-income countries face disproportionately high levels of infectious disease, but low levels of investment in health system capacity due to enduring legacies of colonialism and current economic structures shaped by capitalism and globalization.^20^ Meanwhile, in innovation, the allocation of innovation funding raises questions about who benefits from R&D infrastructure development; for example, do we just enrichen pharmaceutical companies in high-income countries, which disproportionately hire and work for the benefit of privileged groups, when the world invests billions of dollars into innovation? Another challenge that has been made apparent during the COVID-19 pandemic is the inequitable distribution of new technologies, such as vaccines, diagnostic tests, and antimicrobials, once discovered and subsequently brought to market.^21^ The governance challenge for equity is that the global system remains one of self-interested states and profit-driven corporations that always serve their own interests first — even when that behavior contradicts the globality of health threats and prolongs global health crises.

Additionally, while no one is immune to resistant pathogens, different sectors of the population face different risk levels and distributions of infectious disease prevalence and burden. Critical race, class, and gender dimensions are significant here as women and other marginalized groups are more likely to live in poverty, be frontline and informal workers, and face increased risks of infection exposure during pregnancy, abortion, and childbirth.^22^ Overall, key questions about who pays, who gets what, and who is at risk, such as the above, should drive equity considerations in AMR access, conservation, innovation, and IP initiatives.

Table 1 simplifies the inherent complexity of the problem and identifies areas that a pandemic instrument can target to address AMR. Yet, while tools like the this table are helpful to frame problems, they say little about the kinds of policy interventions or approaches that those specific areas need. For the latter, we need a theoretical framework to further analyze the nature of problems and possible solutions.

## 2. Theoretical Framing: Problem Structure and Solution Signals

Theory-driven insights can link governance challenges with potential policy solutions. Here we draw on collective action theory and select examples of how collective action is achieved in biodiversity, climate, and nuclear governance to make potential solutions for AMR more tractable. We focus on the one specific type of collective action problem for AMR most frequently identified in the literature: managing the global common pool of antimicrobial effectiveness. In doing so, we must heed a word of caution when using collective action theory, or any other theory, narrowly like this to conceptualize policy problems and solutions. Primarily, when the dominant framing is one of a common pool resource problem, there is a risk of reducing the multifaceted challenge of AMR to only one dimension of the issue (i.e., conserving and replenishing the common pool resource of antimicrobial effectiveness). However, as section 1 has demonstrated, AMR presents several governance challenges beyond this one aspect.

Considering this limitation, a direct application of collective action theory to AMR can still tease out policy insights to help address the common-pool resource problem that AMR presents, including which design, implementation, or enforcement tools might provide more effective and ethical solutions. By way of background, collective action problems are an especially difficult social problem where the best collective outcome is threatened when individuals follow their immediate self-interests. Common-pool resource challenges are one specific type of collective action problem and existing research has observed that antimicrobial effectiveness resembles a non-excludable but rivalrous common pool resource challenge.^23^

Collective action theory indicates that global common pool resources require governance mechanisms that can change individual self-seeking behaviors — or change the social structures that shape those behaviors — to achieve a collectively beneficial outcome. Specifically, common-pool resource challenges require these kinds of behavior-changing mechanisms across two dimensions: *provision* and *use* of the common pool. In alignment with the governance challenges for AMR described in part 1, the dimension of provision translates into questions about *innovation*, while the dimension of use translates into questions about who has *access* to the resource and who must make a greater *conservation* effort to preserve it.^24^

Techniques used in climate, biodiversity, and nuclear governance offer lessons for managing the global common pool of antimicrobial effectiveness. More specifically, they indicate that a suite of at least 5 design elements can help the pandemic instrument distribute responsibilities and benefits across the governance areas of access, conservation, innovation, and prevention to equitably manage antimicrobial effectiveness as a global common pool resource.

Following the implications of collective action theory, the act of governing the global common pool of antimicrobial effectiveness must inevitably involve decisions about whose interests are counted and prioritized over others, who must change their behavior, what that new behavior is (e.g., “best practices”), and who makes these decisions around access to, conservation of, and innovation for antimicrobials.^25^ Thus, these governance challenges are also inherently political.

In this context, techniques used in climate, biodiversity, and nuclear governance offer lessons for managing the global common pool of antimicrobial effectiveness. More specifically, they indicate that a suite of at least 5 design elements can help the pandemic instrument distribute responsibilities and benefits across the governance areas of access, conservation, and innovation to equitably manage antimicrobial effectiveness as a global common pool resource.

First, *a multi-stakeholder forum* can help inclusively set priorities for access, conservation, and innovation.^26^ The regular multi-stakeholder forums for the Convention on Biological Diversity and the United Nations Framework Convention on Climate Change have helped to ensure inclusivity when deciding governance priorities and distributing responsibilities.^27^ Including a similar global forum within a pandemic instrument could do the same for AMR, while also helping to mobilize and sustain attention on it and other prescient global health issues.^28^

Second, *a science-policy-evidence interface* can help equitably determine best practices for achieving established priorities, so long as it acknowledges that different ways of knowing constitute the global knowledge base. The science and policy interfaces established to curate knowledge on biodiversity and climate issues, the Intergovernmental Science-Policy Platform on Biodiversity and Ecosystem Services (IPBES) and the Intergovernmental Panel on Climate Change (IPCC) respectively, still struggle with representing different understandings of the problems and how best to address them. Including AMR in a pandemic instrumentcould deliver a similar mechanism that can help curate knowledge and confront existing uncertainties for AMR. But learning from these other challenges means recognizing the political challenges associated with this task head on.

Third, *a combination of common and differentiated responsibilities* can help equitably distribute burdens and benefits for AMR.^29^ In climate agreements, the use of common but differentiated responsibilities attempts to achieve a more equitable distribution of burdens and benefits across countries. The principle arises from a recognition of different national capabilities to respond and uneven global histories of exploitation and overuse. For AMR, too, there are parallel cooperative behaviors that all countries need to take to address AMR, such as standardized surveillance and monitoring; but there must also be differentiated responsibilities for other common goals, such as conservation. The latter could mean that while all countries have a common responsibility to use antimicrobials better, high-income countries may have a greater responsibility to restrict their use (conserve) while taking on the bulk of responsibility to replenish the resource (innovation). Lower-income countries, on the other hand, still require greater access to effective antimicrobials, underscoring the need to adapt common global goals to locally specific needs.

Fourth, a pandemic instrument should include *enforcement mechanisms* because there is an inherent risk that countries may free ride off the efforts of others. Enforcement mechanisms are also one of the few design elements proven to affect treaty outcomes in international law.^30^ However, since climate and biodiversity agreements have historically suffered from a lack of enforcement and accountability mechanisms, nuclear technology governance can offer more pertinent lessons here.^31^ For example, the International Atomic Energy Agency has special privileges that allow them to conduct on-site inspections and enact enforcement and accountability measures when states are non-compliant. Similar strategies could be used to bolster global AMR governance, but there are significant political obstacles that may prevent the inclusion of such stringent mechanisms for AMR and other global health issues within a pandemic instrument. The challenge is that states are unlikely to commit to strict rules that they do not think they will meet when costs associated with not following the rules are high. Indeed, others have previously observed an inverse relationship between the ambition of a treaty and the strictness of enforcement mechanisms that states are willing to adopt.^32^ In the case of nuclear technology, however, the intensely high global stakes of any nuclear catastrophe, combined with strong leadership from powerful states during the 1950s, explain why states committed to both ambitious policies and strong enforcement measures in this instance.^33^ One could argue that the high stakes of AMR similarly require strong leadership to deliver ambitious commitments and strong enforcement mechanisms for this global health challenge.

Finally, *an international legal agreement* can unify these elements under a common and coordinating framework. Efforts to manage these other common pool resources benefit from legally enshrined frameworks that foster cooperation, craft incentives, and distribute responsibilities and benefits across countries. AMR currently lacks an equivalent vehicle, but a pandemic instrument could potentially fill this gap if designed to also include AMR.

## 3. Conclusions

AMR is among the leading causes of mortality worldwide, and the governance challenges identified in part 1 offer several potential sites for action that a pandemic instrument could address to mitigate AMR’s global impact. Specifically, we argue that a pandemic instrument must include mechanisms that: (1) equitably address the access gap for antimicrobials, diagnostic technologies, and alternative therapies including other medical countermeasures; (2) equitably conserve antimicrobials to sustain effectiveness and access across time and space; (3) equitably finance the investment, discovery, development, and distribution of new technologies including new antimicrobials and other medical countermeasures; and (4) equitably finance and establish greater upstream and midstream infection prevention and control measures globally.

Existing theoretical work elaborated in part 2 can also be leveraged to generate policy insights for managing the global common pool of antimicrobial effectiveness. Applying collective action theory to the common pool resource problem of AMR indicates that negotiators must carefully consider questions about how burdens and benefits are ethically distributed, including who accesses the common pool resource, whose access gets restricted, and who innovates to replenish it. Past examples from climate, biodiversity, and nuclear governance offer concrete examples for addressing the political considerations associated with these political decisions.

While we still need to generate theory-driven solutions for the full range of collective action problems for AMR the pandemic treaty offers a much-needed opportunity to swiftly deliver global regulations on AMR. With the pandemic instrument, we can start turning “collective action problems” into “collective action solutions” for AMR. Given the urgency of the situation, there is no more time to wait.

## Note

This work was supported by the Canadian Institutes of Health Research [#149542], the Social Sciences & Humanities Research Council [#895-2022-1015], the Wellcome Trust [222422/Z/21/Z], and internal funding from the International Research Strategy Working Group at the University of Cambridge. Isaac Weldon is supported by the Canadian Institutes of Health Research. Timo Minssen’s research for this contribution was supported by a Novo Nordisk Foundation grant for a scientifically independent Collaborative Research Programme in Biomedical Innovation Law (Grant agreement number NNF17SA0027784). The funding bodies were not involved in the study design, data collection, analysis, interpretation or writing.

Timo Minssen serves as expert advisor to WHO Digital Health & COVID-19 Law & Ethics Committees, and the Oslo Medicines Initiative. He is also a Senior Advisor at the Swedish law firm X-Officio Advokat AB, and a scientifically independent IP Advisory Board Member of the Danish life science company Chr. Hansen A/S. Steven J. Hoffman is the Vice-President of Corporate Data & Surveillance at the Public Health Agency of Canada (PHAC) and previously served as the Scientific Director of the Institute of Population & Public Health at the Canadian Institutes of Health Research (CIHR). The views expressed in this article are those of the authors and do not necessarily reflect those of their affiliated institutions.
